# Transcriptomes of post-mitotic neurons identify the usage of alternative pathways during adult and embryonic neuronal differentiation

**DOI:** 10.1186/s12864-015-2215-8

**Published:** 2015-12-23

**Authors:** Alexandra Tallafuss, Meghan Kelly, Leslie Gay, Dan Gibson, Peter Batzel, Kate V. Karfilis, Judith Eisen, Kryn Stankunas, John H. Postlethwait, Philip Washbourne

**Affiliations:** Institute of Neuroscience, University of Oregon, Eugene, OR USA; Institute of Molecular Biology, University of Oregon, Eugene, OR USA; Current address: Vollum Institute, Oregon Health and Science University, Portland, OR USA

**Keywords:** 4tU-labeling, Transcriptome, Differentiation, Zebrafish, Uprt, Elavl3, Differential expression, Neuron

## Abstract

**Background:**

Understanding the mechanisms by which neurons are generated and specified, and how they integrate into functional circuits is key to being able to treat disorders of the nervous system and acute brain trauma. Much of what we know about neuronal differentiation has been studied in developing embryos, but differentiation steps may be very different during adult neurogenesis. For this reason, we compared the transcriptomes of newly differentiated neurons in zebrafish embryos and adults.

**Results:**

Using a 4tU RNA labeling method, we isolated and sequenced mRNA specifically from cells of one day old embryos and adults expressing the transgene *HA-uprt-mcherry* under control of the neuronal marker *elavl3*. By categorizing transcript products into different protein classes, we identified similarities and differences of gene usage between adult and embryonic neuronal differentiation. We found that neurons in the adult brain and in the nervous system of one day old embryos commonly use transcription factors - some of them identical - during the differentiation process. When we directly compared adult differentiating neurons to embryonic differentiating neurons, however, we found that during adult neuronal differentiation, the expression of neuropeptides and neurotransmitter pathway genes is more common, whereas classical developmental signaling through secreted molecules like Hedgehog or Wnt are less enriched, as compared to embryonic stages.

**Conclusions:**

We conclude that both adult and embryonic differentiating neurons show enriched use of transcription factors compared to surrounding cells. However, adult and embryonic developing neurons use alternative pathways to differentiate. Our study provides evidence that adult neuronal differentiation is distinct from the better characterized embryonic neuronal differentiation process. This important insight and the lists of enriched genes we have identified will now help pave the way to a better understanding of the mechanisms of embryonic and adult neuronal differentiation and how to manipulate these processes.

**Electronic supplementary material:**

The online version of this article (doi:10.1186/s12864-015-2215-8) contains supplementary material, which is available to authorized users.

## Background

During early stages of vertebrate embryonic development, a variety of signaling pathways, including BMP, Wnt, Fgf and Shh are necessary for neuronal induction and patterning [1–3], while Notch signaling is critical to establish cell identity [4]. Vertebrate embryonic neurons are born in proliferative zones often located near ventricles. Cells that are determined to become neurons must differentiate and migrate to their destinations where they mature into functional neurons. This process depends both on extrinsic factors, such as signaling molecules, and on intrinsic factors, such as transcription factors.

Neurons proliferate throughout the entire life of vertebrates. In adult mammals, proliferation in the brain appears to be most prominent in the subventricular and the subgranular zones of the telencephalon [5, 6], but additional neurogenic zones have been reported in other brain regions in non-mammalian vertebrates (see for review [7]). In contrast to mammals, in teleost fish neuronal progenitor proliferation occurs at a much higher rate and proliferation zones have been identified in many different brain regions, not only located near ventricles but also in distinct regions such as the olfactory bulb, dorsal telencephalon, hypothalamus, preoptic area, hypothalamus, optic tectum, and cerebellum [8–10]. Some molecular factors are common during both adult and embryonic neurogenesis, for instance, adult and embryonic radial glial cells that act as neuronal progenitors [11] express glial acidic fibrillary protein (GFAP) or S100 β. Recent research has focused mostly on proliferation and neurogenesis zones, leaving the molecular identity of pathways involved during proliferation and neuronal differentiation under-explored. Finding the common and distinct traits that characterize adult and embryonic neuronal development would help answer questions as to how new neurons migrate, mature and, finally, how these newly made neurons incorporate into an existing neural network.

We used 4tU-labeling, a method based on expression of the introduced transgene *uracil-phosphoribosyl-transferase* (*uprt*) [12, 13], to identify the transcriptome of newly made neurons in developing and adult zebrafish (see 4tU flow-chart shown in Additional file 1). Uprt expression allows cells to utilize 4-thiouracil (4tU) as a substrate for making 4t-UTP, which is then incorporated into newly transcribed RNA. Importantly, only cells expressing the transgene are able to incorporate 4tU [14]. This method has the advantage of avoiding manual dissections or fluorescence activated cell sorting and preventing any gene expression changes induced by stress (see [15]). By expressing HA-Uprt-mCherry under the control of the *elavl3:gal4* driver line [16], we were able to specifically isolate RNA from differentiating neurons in both adult and embryonic zebrafish [17–19]. ELAV-like neuron-specific RNA binding protein 3 (Elavl3) is required for correct differentiation and development of the nervous system [20].

We compared the transcriptomes of *elavl3*-positive differentiating neurons in the embryonic and adult nervous system and characterized expression of highly enriched or depleted genes. We found that proteins involved in nucleic acid binding are more frequent in differentiating neurons compared to the total of all neurons in the nervous system in both embryos and adults. However, our results suggest that the differentiation process of neurons in adults and embryos is vastly different both at the gene level and the pathway level. These results are significant because they start to fill a large gap in our knowledge regarding the differentiation and maturation of neurons during adult neurogenesis and regeneration.

## Results and discussion

### The HA-Uprt-mcherry transgene is expressed in neurons in the adult zebrafish brain

**Fig. 1:**
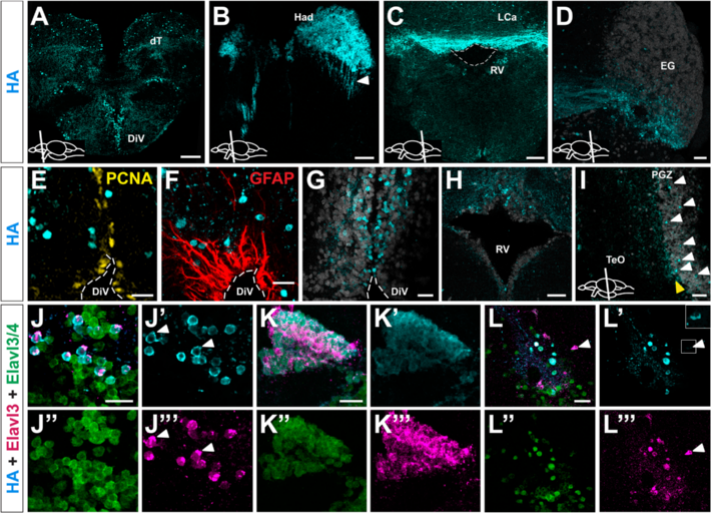
Distribution of HA-Uprt-mCherry transgene expression on cross sections of adult zebrafish brain. **a** Section showing HA labeling (*cyan*) in the dorsal telencephalon (dT) and the diencephalic ventricle (DiV). **b** High magnification of the dorsal habenula (Had) in the anterior diencephalon, with HA labeling in cells and projections (white arrowhead) from the Had to the interpeduncular nucleus (IPN) in the mesencephalon. **c** Section through the cerebellum showing HA-localization near the rhombencephalic ventricle (RV, Lobus caudalis cerebelli (LCa)). **d** HA-expression in the ventral half of the eminentia granularis (EG) and axon projections. Elavl3/4 labeling (*white*) marks most cells of the EG. **e** Co-labeling of HA and the proliferation marker PCNA (*yellow*) in a high magnification of the diencephalic ventricle (DiV) shows no overlap. **f** HA-labeling and the glial marker GFAP (*red*) are not co-expressed in the same cells. **g** HA-positive cells in the vicinity of the diencephalic ventricle. Elavl3/4 labeling (white) partially overlaps with HA labeling (*cyan*) but shows a much broader expression domain than HA labeling. **h** Most HA-positive neurons are located in the dorsal part of the rhombencephalic ventricle (RV). The ventricle is outlined by Elavl3/4 labeling (*white*). **i** Some HA-positive neurons are located within the periventricular gray zone (PGZ, white arrowheads). Many HA-labeled neurons are located at the margin of the PGZ and the optic tectum (*TeO, yellow arrowhead*). Elavl3/4 labeling marks most cells of the PGZ. **j** High magnification of the diencephalic ventricular zone showing co-expression of HA (*cyan*) and endogenous Elavl3 (*magenta*) within the population of Elavl3/4-positive neurons (*green*). **k** Overlapping expression of HA, Elavl3 and Elavl3/4 in the dorsal habenula. **l** Ventrally located nucleus in the hypothalamus shows that all Elavl3-positive neurons co-express HA-Uprt-mCherry (*white arrowhead and inset*). Cartoons in a-d and i show lateral views of adult zebrafish brain indicating the level of the individual cross sections. e-g and i. Sections show a similar level as in **a** or **c**, respectively. The panels **j’, j”, j”’, k’, k”, k”’** and **l’, l”, l”’** show single channels. Scale bars represent 100 um in a; 40 um in b, g; 150 um in c; 45 um in d, h; 25 um in e, f; 35 um in i; 20 um in h, k; and 30 um in l

Although the *elavl3* promoter is commonly used as a pan-neuronal driver for transgene expression in developmental studies [17, 21–23], its expression pattern in the adult brain has not been well characterized. We examined expression of the fusion protein HA-Uprt-mCherry by immunohistochemistry (IHC) for the epitope HA (Fig. 1) or the fluorescent marker mCherry (not shown) on cross sections of brains from 9 month old transgenic adult zebrafish. Results showed robust expression in distinct regions within the forebrain, midbrain and hindbrain (Fig. 1a-d).

In the adult forebrain, HA-Uprt-mCherry was distributed in a salt-and-pepper pattern throughout the olfactory bulb and the dorsal telencephalon (Fig. 1a). We found many cells expressing HA-Uprt-mCherry near the telencephalic and diencephalic ventricle (Fig. 1a) and the telencephalic surface. This distribution reflects the expected location of post-mitotic neurons in the proximity of proliferating zones, as the telencephalon of teleosts forms by eversion, such that the proliferative periventricular zones are not only present internally but also cover the outer surface of the telencephalon [7]. In addition to proliferative zones, the dorsal habenula (Had, Fig. 1b), showed robust expression of the HA-Uprt-mCherry transgene. We noticed a size difference between the left and the right HA-Uprt-mCherry labeling of the dorsal habenula, consistent with previously described left-right asymmetry of the epithalamus in the adult zebrafish brain [24]. Interestingly, axonal projections originating from the dorsal habenula and targeting the ventrally located interpeduncular nucleus were strongly labeled (Fig. 1b, arrowhead), suggesting that HA-Uprt-mCherry is expressed in differentiating neurons and their projections.

In brain regions posterior to the forebrain, the expression of HA-Uprt-mCherry near ventricles appeared to be more restricted. We found a larger number of HA-Uprt-mCherry-positive cells in the vicinity of ventricular zones of the forebrain (Fig. 1g) than in the more caudally located rhombencephalic ventricle (RV, Fig. 1h), where HA-Uprt-mCherry-positive cells are more sparsely distributed around the ventricle. However, many cells were labeled for HA-Uprt-mCherry dorsal to the RV across the corpus cerebelli, lobus caudalis cerebelli (LCa) and laterally in the eminentia granularis (EG), (Fig. 1c). In a slightly more posterior section of the cerebellum, HA-positive neurons appeared in a subset of cells located in the ventral part of the EG (Fig. 1d), with projections extending to or coming from more medial parts of the cerebellum.

We next focused on determining whether the HA-Uprt-mCherry protein expression pattern was indicative of newly differentiated neurons. Therefore, we first compared HA-Uprt-mCherry expression with proliferation and glial cell markers. We expected HA-Uprt-mCherry and the pan-neuronal marker Elavl3/4, an antibody that recognizes both Elavl3 and Elavl4 proteins, to be expressed in post-mitotic neurons [25]. The proliferating cell nuclear antigen (PCNA, yellow) was expressed in cells outlining the ventricle (Fig. 1e, DiV outlined in white). HA-Uprt-mCherry-expressing cells were located at about two cell diameters distance from the ventricle, as would be expected for newly generated neurons that are born in proliferative zones and migrate to their target positions. HA-Uprt-mCherry expression did not overlap with PCNA, suggesting that HA-Uprt-mCherry, similar to Elavl3 (not shown), is not expressed in proliferative or neurogenic cells. Further, HA-Uprt-mCherry was restricted to neuronal cells but not glial cells, as confirmed with the glial cell marker (GFAP, red, DiV outlined in white) (Fig. 1f). These results suggest that HA-Uprt-mCherry, similar to Elavl3 and Elavl3/4 proteins (not shown, [25]), is expressed in post-mitotic neurons and not in other brain cells, including glia, demonstrating specificity.

Next, we tested whether the expression of HA-Uprt-mCherry recapitulated that of endogenous Elavl3 and/or Elavl3/4 proteins. We compared HA-labeling to Elavl3/4-labeling in different parts of the brain (Fig. 1g-i). The observation that Elavl3/4 labeled many more cells than HA was also true for other brain regions, such as the ventricles of the forebrain (Fig. 1g), the cerebellum (Fig. 1h) and the periventricular gray zone (PGZ) in the optic tectum (Fig. 1i). While we found strong expression of Elavl3/4 in the proliferation zone of the PGZ, we found only some cells expressing HA-Uprt-mCherry within the PGZ and many HA-positive neurons at the border of the PGZ and in the optic tectum (Fig. 1i, marked by white and yellow arrowheads, respectively). We conclude that the transgene *HA-uprt-mcherry* is expressed in a subset of Elavl3/4-positive cells, presumably specifically in Elavl3-positive cells.

We then examined whether all cells expressing HA (cyan) also expressed Elavl3 (magenta), confirming the specificity of transgene expression (green, Fig. 1j-l). Zebrafish Elavl3 protein expression has not been described in the central nervous system of zebrafish so far; therefore we compared the Elavl3 expression pattern to the established neuronal marker Elavl3/4 (see ZFIN, Ab1-elavl, http://zfin.org/action/marker/view/ZDB-ATB-081003-2). In the diencephalic ventricle (Fig. 1j) and the dorsal habenula (Fig. 1k) all cells were co-labeled with Elavl3 (magenta) and HA (cyan) while Elavl3/4 (green) was more broadly expressed, suggesting that HA-Uprt-mCherry largely recapitulated endogenous Elavl3 protein expression and not the broader Elav3/4 pattern. Further, in one ventral cluster of cells in the hypothalamus, we found that HA-Uprt-mCherry transgene expression was in all Elavl3-positive cells although weakly in some neurons (Fig. 1l, arrowheads, see higher magnification in inset). We conclude that Elavl3-positive neurons co-express HA-Uprt-mCherry, providing evidence that our transgene is expressed in differentiating neurons.

### 4tU-labeled mRNA reveals neuronal gene expression in adult brain

**Fig. 2:**
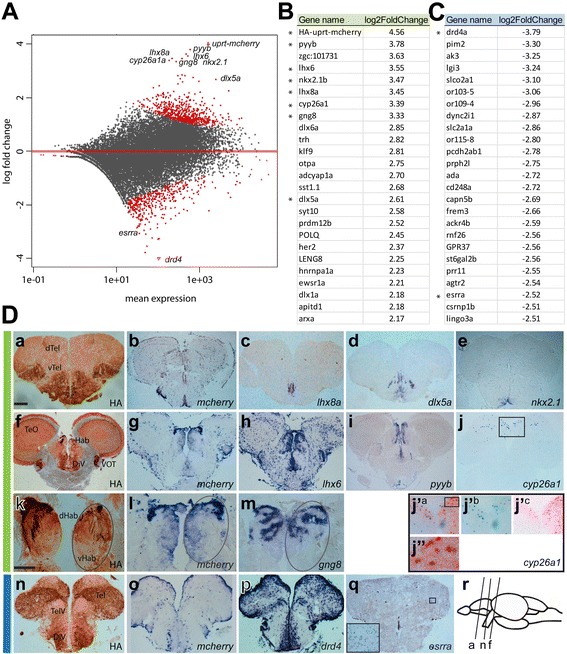
Data analysis and verification of 4tU-labeled mRNA isolated from adult zebrafish brain. **a** Distribution plot showing the log fold change of individual reads compared to the mean expression using DESeq2. The red dots indicate significantly enriched or depleted genes (padj < 0.1). Some labeled enriched or depleted genes were analyzed for their expression pattern (see d). **b** List of the most-enriched genes (*green*) and (**c**) most depleted genes (*blue*) and their log2-fold change compared to total mRNA expression levels. Asterisks mark genes used for expression studies. **d** Cross sections of transgenic adult zebrafish brains labeled with HA labeling (*a, f, k, n*) or in situ probes of enriched genes (*b-e, g-j, l, m* green bar) as indicated on each image. j’^a-c^ and j” are higher magnifications of boxes shown in j and j’, respectively. j’^b^ and j’^c^ show *cyp26a1* in situ hybridization and HA IHC labeling, respectively. j’^a^ and j” show *cyp26a1* in situ hybridization and HA IHC labeling. Expression of depleted genes (*p,q*, blue bar). Cartoon (in *r*) showing the corresponding sections in a, f and n. dTel, dorsal Telencephalon, vTel, ventral Telencephalon, Hab, habenula, dHab, dorsal Hab, vHab, ventral Hab, TeO, optic tectum, DiV, diencephalic ventricle, VOT, ventrolateral optic tract, TelV, telencephalic ventricle. Scale bars represent 100 um in d*a-j, n,q* and 65 um in d*k-m*

After verifying the protein distribution of HA-Uprt-mCherry in the adult brain, we felt confident in analyzing the transcriptome of this identified neuronal population. We treated 9 month old adult female and male zebrafish for four hours with 4tU by intraperitoneal injection, recovered brains and purified 4tU-labeled mRNA. We found that 606 genes were statistically significantly enriched and 406 genes were depleted in the adult brain 4tU-labeled mRNA samples compared to total mRNA, with an adjusted p-value (padj) less than 0.1 (red dots, Fig. 2a). In the following text, we call these significantly differentially expressed genes “enriched” or “depleted,” respectively. A list of the most enriched and depleted genes is shown in Fig. 2b. The full data set can be found in Additional file 2. As expected, the transgene *HA-uprt-mcherry* with a log2-fold change of 4.56 appears on top of the list of all enriched genes (green). This result confirms that we were able to specifically target and isolate mRNA from cells expressing the enzyme HA-Uprt-mCherry.c

To verify that the identified genes are expressed in cells positive for the transgene HA-Uprt-mCherry, we performed in situ hybridization (ISH) and immunohistochemistry (IHC) on brain sections of transgenic zebrafish (Fig. 2d). We randomly chose seven genes that are highly enriched with a log2-fold change of at least 2.5 (denoted by asterisks in Fig. 2b) and compared their expression pattern to *mcherry* expression and/or anti-HA IHC (Fig. 2d). As expected, we found that the expression pattern of all seven enriched genes overlapped with HA-Uprt-mCherry (Fig. 2 d*a,f,k,n*) in neurons. HA antibody labels neurons, similar to ISH, and also neuropil, making HA-labeling appear more broad.

In the adult telencephalon, the protein distribution of HA-Uprt-mCherry was located in the vicinity of the diencephalic ventricle, the telencephalic surface and in neurons throughout the forebrain in a sparse distribution (Fig. 2d*a*, d*n*). In addition, we found strong labeling of the neuropil in the ventral telencephalon, which was identified by protein but not transcript labeling. The transcript *mcherry* was expressed in overlapping regions (Fig. 2d*b*). In a similar section, the expression of the LIM homeobox transcription factor (TF)-encoding gene *lhx8a* was restricted to the ventral ventricle (Fig. 2d*c*), whereas the distal-less homeobox TF-encoding gene *dlx5a* was expressed near the ventricle and in additional clusters lateral to the ventricle (Fig. 2d*d*). Expression of the NK2 homeobox TF-encoding gene *nkx2.1* was restricted to the ventral-most part of the ventricle (Fig. 2d*e*). In a more posterior section of the telencephalon, we found HA labeling in part of the habenula (Hab), the ventrolateral optic tract (VOT), the optic tectum (TeO) and near the diencephalic ventricle (DiV, Fig. 2d*f*). We found a similar distribution of *mcherry* transcript expression (Fig. 2d*g*). The LIM homeobox TF-encoding gene *lhx6* (Fig. 2d*h*) and the neuropeptide gene *pyyb* (Fig. 2d*i*) were expressed in similar domains as *mcherry* (Fig. 2d*h*, d*i*). However, *lhx6* was more broadly expressed in neurons throughout the telencephalon (Fig. 2d*h*). In contrast, *cyp26a1,* which encodes a retinoic acid metabolizing enzyme cytochrome P450 superfamily member, was expressed sparsely in the dorsal part of the diencephalon (Fig. 2d*j*). We verified co-expression of all enriched genes with HA-Uprt-mCherry by two-color ISH/IHC labeling. As an example we show that *cyp26a1*-positive cells (blue) were co-labeled with HA-Uprt-mCherry protein (magenta) in some but not all cells (Fig. 2dj^a-c^, and j” showing a magnification of the inset in j’^a^). This result suggests that 4tU-labeling is sensitive to transcripts that may be expressed in only a few cells. Further, we localized expression of *mcherry* mRNA to the dorsal habenula (Fig. 2d*l*), consistent with the HA-Uprt-mCherry protein distribution (Figs. 2d*k*, 1b). The guanine nucleotide binding protein (G protein) *gng8*, a known marker of the habenula, was an enriched gene (Fig. 2a, b) and overlapped well with *mcherry* expression (Fig. 2d*m*). We conclude that all genes tested are expressed in neurons that overlap with the transgene HA-Uprt-mCherry. This result suggests that our procedure isolated preferentially transcripts expressed in cells containing HA-Uprt-mCherry, which allowed us to identify transcripts enriched specifically in differentiating neurons.

We also examined the expression patterns of two depleted genes (see Fig. 2c): *drd4* and *esrra*. The dopamine receptor *drd4* showed strong expression in the forebrain (Fig. 2d*p*) that partially overlapped, but had much broader expression, than HA-Uprt-mCherry protein and *mcherry* transcript (Fig. 2d*n,o*). We found ubiquitous expression for the estrogen receptor *esrra* (Fig. 2d*q*) with spotted labeling (Fig. 2d*q* inset) and more restricted expression in the olfactory bulb (not shown). In general, HA-Uprt-mCherry appeared more restricted than the expression of the tested depleted genes, leading to a higher number of transcripts in total mRNA compared to mRNA isolated from HA-Uprt-mCherry-positive neurons.

In summary, we specifically isolated and verified mRNA transcripts of even infrequently expressed genes from HA-Uprt-mCherry-positive neurons in the adult zebrafish brain.

### Gene ontology analysis shows enrichment and depletion of specific types of genes in adult brain

**Fig. 3:**
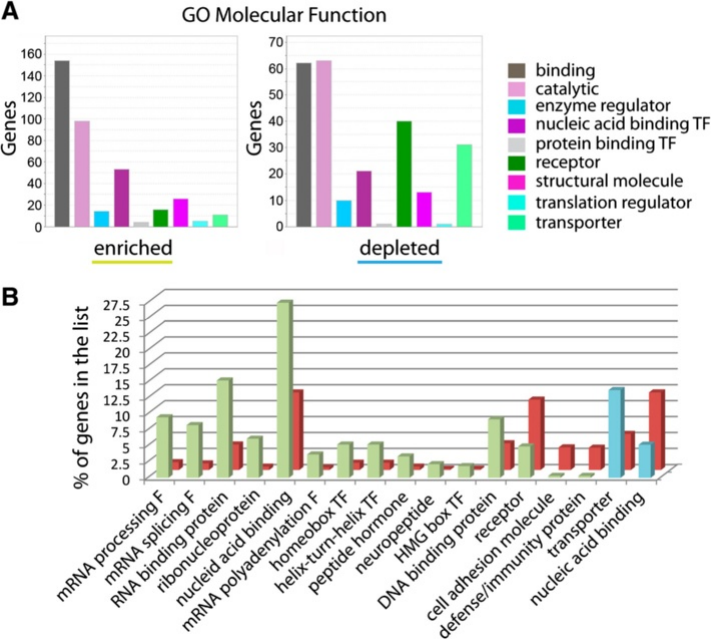
Molecular function and protein classes for enriched and depleted genes from adult zebrafish brain. **a** Graphs showing significantly enriched or depleted genes with padj < 0.05 categorized into different classes of molecular function using Panther gene ontology tools. The “number of genes” falling into each protein class is indicated on the Y-axis. **b** Overrepresentation graph of enriched (*green*) and depleted (*blue*) genes (padj < 0.05) showing the percent of genes in the list compared to the reference gene list (*red*) and categorized for protein class

We used Protein Analysis Through Evolutionary Relationships (Panther) analysis tools [26, 27] to identify the most relevant molecular function categories of enriched and depleted genes, respectively (padj < 0.05, Fig. 3a). Panther output charts give an overview of the molecular function of the genes in the list and one gene can appear in multiple molecular function categories. We analyzed a total of 332 enriched genes, resulting in 381 function hits. We found that the majority of enriched genes fell into the gene ontology (GO) class “binding” (45 % of total differentially expressed genes), which includes nucleotide, chromatin, ion, lipid and antigen binding. Additional highly represented categories were “catalytic” (29 %) and transcription factors (TF, 15 %) while other categories, such as structural molecules, regulator, protein binding TF, receptor and transporter were not strongly represented (under 7 %). The most common GO term categories in the depleted gene list (223 genes analyzed with 242 function hits) were “binding” (28 %) and “catalytic” (28 %), similar to the enriched genes (Fig. 3a). However, in contrast to enriched genes, receptors (18 %) and transporter activity (13 %) were more frequent in the depleted list. Other categories were represented in less than 4 % of all genes. We conclude that “binding” appears to be a relevant function in newly made neurons, while receptors and transporters are expressed less than in the rest of the brain.

To find the protein classes that are strongly represented in differentiating neurons, we used the Panther overrepresentation test, which compares the proportion of genes assigned to a functional class between a given gene list (Fig. 3b, enriched genes in green, depleted in blue) and a reference gene list (Fig. 3b, red). As a reference gene list we used all zebrafish genes in the database (Panther Overrepresentation test release 20150430, including 25357 mapped gene IDs). Using the list of enriched genes, protein classes that were overrepresented included proteins involved in mRNA processing and splicing, nucleic acid, DNA- and RNA- binding and different classes of transcription factors (Fig. 3b, green, *p* < 0.05, Bonferroni correction, for p-values see Additional file 3) compared to the reference gene list. This result is consistent with actively differentiating cells.

Other protein classes expected in more mature neurons, such as receptors and cell adhesion molecules, were represented less frequently than the reference gene list. In 332 enriched genes (padj < 0.05), we did not find genes typically expressed in proliferating cells or mature neurons, suggesting that our samples were not significantly contaminated by other cell types. Within the depleted genes (Fig. 3b, blue), transporter proteins were more frequently represented compared to the reference gene list, while nucleic acid binding proteins were under-represented. Our data suggest that in the adult zebrafish brain, binding proteins, RNA-modifying proteins and neuropeptides are preferentially expressed during neuronal differentiation.

### 4tU-labeled mRNA reveals gene expression in embryonic brain

We next asked whether genes similar to the ones enriched in adult zebrafish brain are also enriched during neuronal differentiation in zebrafish embryos. We prepared RNAseq libraries from one day old embryos, a stage that is often used for expression pattern analysis because the developing nervous system has advanced to a morphology that allows identification of different brain anlage substructures [28]. Embryos were incubated with 4tU in embryo medium starting at 22 h post fertilization (hpf), sacrificed after 4 h of incubation and used without any further dissection for 4tU-labeled RNA purification.

**Fig. 4:**
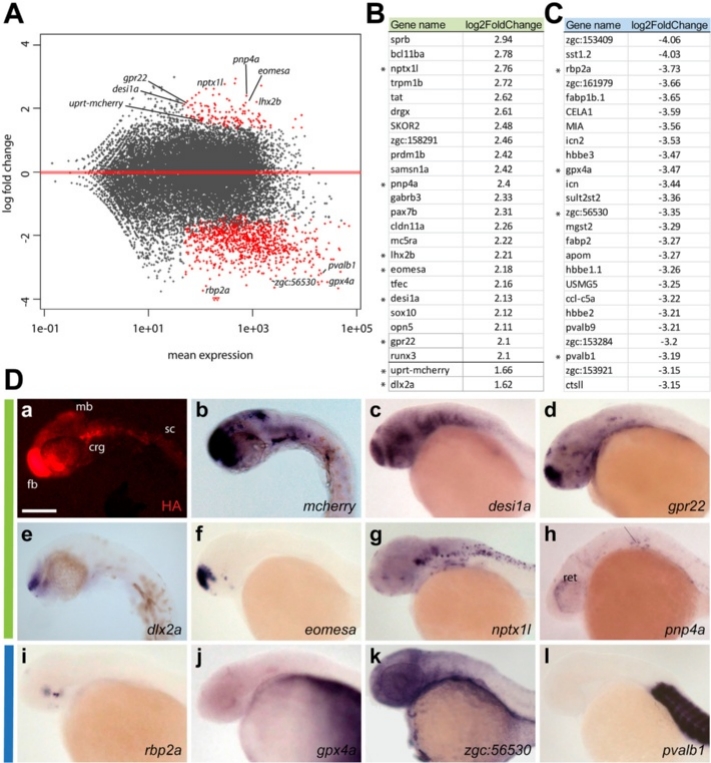
Data analysis and expression patterns of 4tU-labeled mRNA transcripts isolated from whole embryos. **a** Distribution plot showing the log fold change of individual reads compared to the mean expression using DESeq2. The red dots indicate significantly enriched or depleted genes. **b** List of the top enriched (*green*) and (**c**) depleted (*blue*) genes and their log2-fold change compared to total mRNA expression levels. Genes used for expression study are marked with asterisks. In addition *uprt-mcherry* and *dlx2a* are shown to demonstrate enrichment for the transgene and a gene common with adult purified mRNA, respectively. **d** In situ hybridization of 24–26 hpf embryos, lateral view showing enriched (*b-h*, *green bar*) or depleted genes (*i-l, blue bar*). d*c,d,f-l* show images published by The Zebrafish Model Organism Database (ZFIN) [47, 48]. fb, forebrain, mb, midbrain, crg, cranial ganglia, sc, spinal cord. Scale bar represents 100 um

Results identified 154 statistically significantly enriched genes and 992 depleted genes compared to total mRNA isolated from the same embryos (Fig. 4a). Red dots in Fig. 4a represent significantly enriched and significantly depleted genes (*p*-adjusted value < 0.1). In Fig. 4b and c, we list the top enriched and depleted genes. The full list of genes appears in Additional file 4. The transgene *HA-uprt-mcherry* was 1.66 log2 fold enriched. Because Uprt-mCherry-HA is expressed in many neuronal cells at this stage, the differences between 4tU-labeled mRNA and total mRNA is not expected to be very high [29].

To further validate the specificity of the 4tU labeling method, we examined the expression patterns of some of the genes with high or intermediate log-fold change in 22–26 hpf embryos by ISH (marked by asterisks in Fig. 4b). Uprt-mCherry protein (Fig. 4 d*a*) and transcript (Fig. 4d*b*) were expressed almost exclusively, but broadly, in the nervous system. The desumoylating isopeptidase *desi1a* (Fig. 4d*c*) was expressed in tissues very similar to *uprt-mcherry* in the forebrain, retina, midbrain, hindbrain and cranial ganglia. In contrast, distal-less homeobox TF *dlx2a* and eomesodermin homolog *eomesa* showed expression restricted to the forebrain (Fig. 4d*e,f*). The G protein-coupled receptor *gpr22* was strongly expressed in the forebrain, epiphysis, olfactory bulb, cranial ganglia and heart tube (Fig. 4d*d*). In contrast, neuronal pentraxin-like gene *nptx1l* lacked expression in the forebrain, but was strongly expressed in the cranial ganglia, spinal cord and in the pharyngeal arches (Fig. 4d*g*). The purine nucleoside phosphorylase gene *pnp4a* showed expression in the retina and in some pigment cells (Fig. 4d*h*). The expression patterns of all these genes overlapped well with at least some regions expressing HA-Uprt-mCherry.

We then examined the expression of depleted genes (Fig. 4d*i-l*). Results showed that RNA polymerase *rbp2a* was expressed in a cluster of cells in the ventral retina (Fig. 4d*i*), presumably retinoic acid-synthesizing enzyme expressing cells [30], whereas the glutathione peroxidase gene *gpx4a* was expressed only in the yolk syncytial layer and not in the nervous system (Fig. 4d*j*). The cystatin 14a.2 gene *cst14a.2* (formerly known as *zgc:56530*) was detected in axial vasculature, epidermis, hatching gland and also showed expression in the yolk syncytial layer (Fig. 4d*k*). Further, we found that parvalbumin *pvalb1* was strongly expressed in the muscle (Fig. 4d*l*). These results showing that depleted genes are not expressed in *elavl3*-expressing cells confirm the specificity of enriched genes to tissues that express the HA-Uprt-mCherry transgene.

### Gene ontology analysis shows that transcription factor-encoding genes are enriched in the embryonic nervous system

**Fig. 5:**
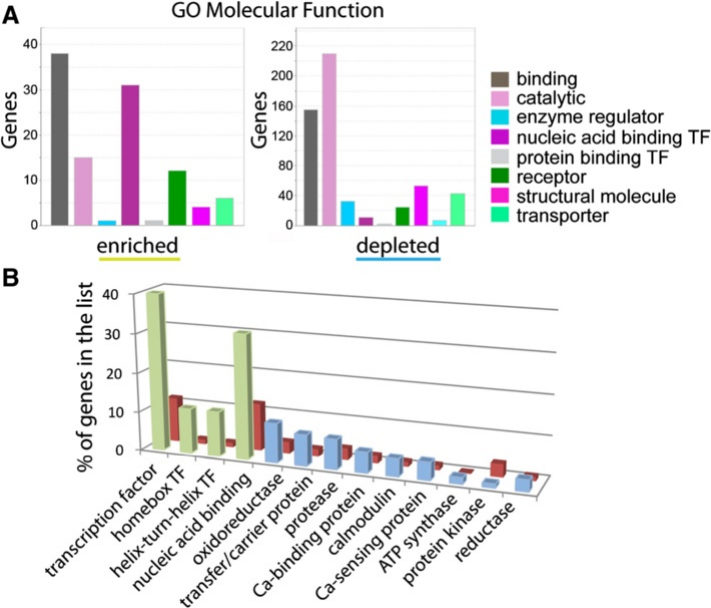
Graphs showing molecular function and protein class for enriched and depleted genes from zebrafish embryos. **a** Gene Ontology graphs showing the molecular function of significantly enriched or depleted genes with padj < 0.05. The Y-axis shows the number of genes in each category. **b** Overrepresentation graph of enriched (*green*) and depleted (*blue*) genes (padj < 0.05) showing the percent of genes in the list compared to a reference gene list (*red*) and categorized for protein class

We examined the molecular function (Fig. 5a) of enriched genes in embryonic brains (79 genes analyzed, padj < 0.05) using Panther software. We found a high number of genes correlating with the GO term “binding” (48 %), nucleic acid binding TF (47 %), “catalytic” (18 %) and receptors (15 %). In the 637 depleted genes, we found that most genes were linked to “catalytic” (36 %) and to a lesser extent “binding” (24 %) functions. Other categories were represented to a lower extent (<7 %). Results suggest that nucleic acid binding TF activity appears to be critical for differentiating neurons compared to other cells in the embryo (47 % in enriched vs 1 % in depleted).

We then examined whether TFs and binding proteins are overrepresented in embryonic enriched genes (Fig. 5b, green, for *p*-values see Additional file 3). Nucleic acid binding proteins and TFs in general and in particular homeobox and helix-turn-helix TFs were found in much higher percentages than expected when compared to the reference gene list (red). In the depleted gene list (Fig. 5b, blue), consisting of genes originating from many different tissues, we found several protein classes moderately overrepresented compared to the reference gene list, for instance transfer/carrier proteins and Calcium-related proteins. This data identifies TFs as the most relevant protein class in embryonic differentiating neurons.

In summary, we identified and verified genes significantly enriched and depleted in differentiating neurons in the developing embryo, many of which are nucleic acid binding proteins and are likely important during neuronal differentiation.

### Adult and embryonic differentiating neurons express distinct suites of genes

**Fig. 6:**
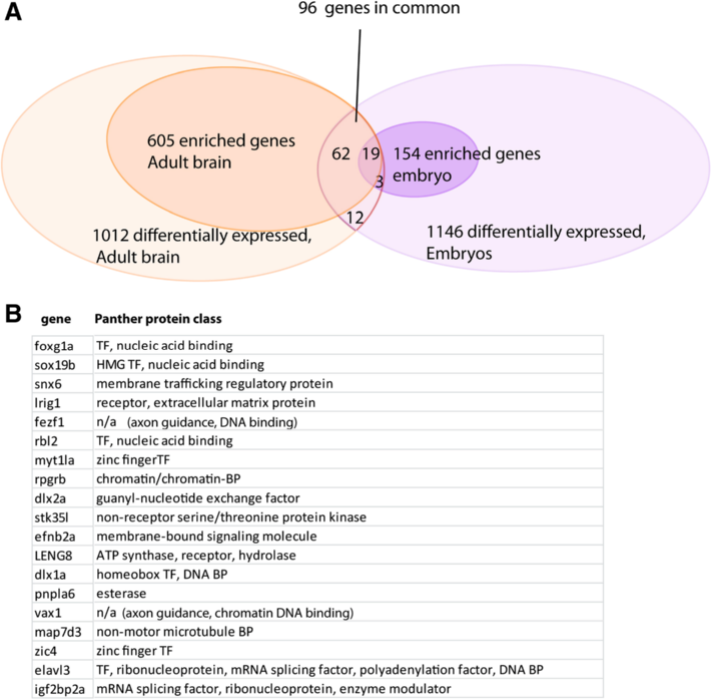
Common genes found in adult brain and embryonic nervous system. **a** Venn diagram showing the distribution of all statistically significant genes for adult brain and embryonic nervous system. Enriched genes are a subset for both adult and embryonic sets. 19 genes are significantly enriched in both adult brain and embryonic nervous system (**b**). Table with a list of the 19 enriched genes common to both adult brain and embryonic nervous system

To find genes common to neuronal differentiation in both adults and embryos, we directly compared all statistically significant genes (padj < 0.1; enriched and depleted) that were differentially expressed in adults with differentially expressed genes in embryos. Of the 1012 differentially expressed genes from adult brain and the 1146 differentially expressed genes from embryos, only 96 were in common (Fig. 6). Further, when we restricted analysis to enriched genes from both cell populations (605 in adult, 154 in embryos), we found just 19 enriched genes in common (see list, Fig. 6), suggesting that these genes are used for similar processes during neuronal differentiation at both life stages. This number represents only about 2.5 % of the total number of enriched genes found in both adult and embryonic differentiating neurons, implying that adult and embryonic neuronal differentiation might have substantial differences in gene usage. This small number of common genes is consistent with the idea that there may be important differences in neuronal differentiation at these two developmental stages: the embryonic nervous system is forming de novo, while neurons differentiating in the adult are integrating into an already existing functional nervous system.

To identify genes that are differentially expressed between neurons of the adult brain and the embryonic nervous system, we directly compared the number of genes for the purified samples between adults and embryos. Genes enriched in embryos (vs adults) were then filtered for neuronal expression, using nervous system-specific genes obtained from ZebrafishMine (http://www.zebrafishmine.org/begin.do) and Panther Classification system [27] (see Additional file 5). This analysis resulted in 3111 genes enriched and 559 decreased in adult versus embryo (see Additional file 6).

**Fig. 7:**
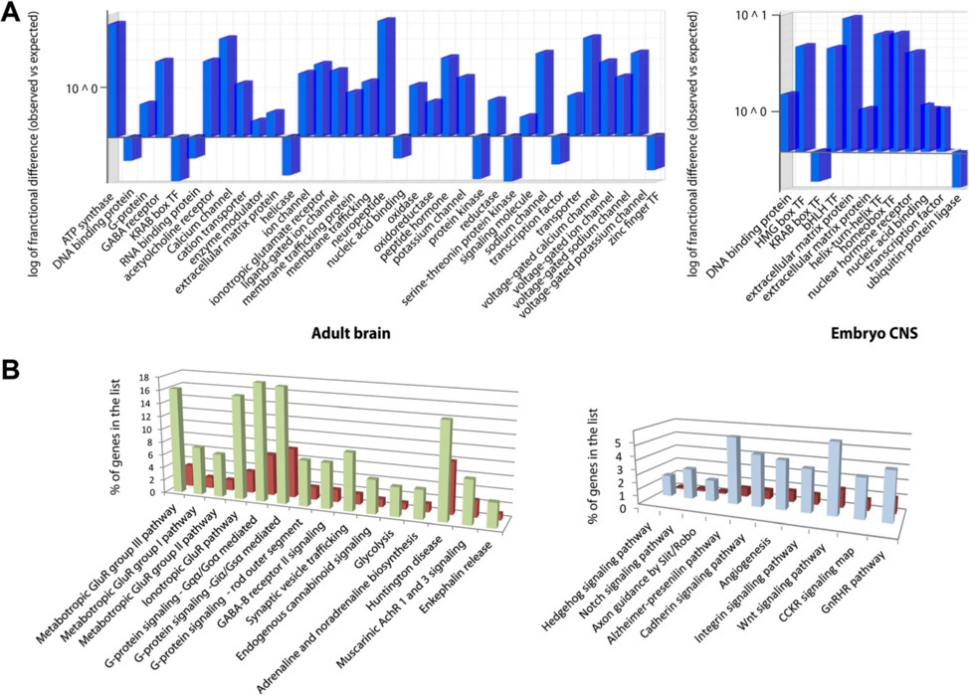
Transcriptome differences between adult brain and embryonic nervous system. **a** Graphs showing log of fractional difference between observed and expected protein classes of enriched transcripts in adult brain and embryonic nervous system. **b** Graphs showing overrepresented pathways in adult (*green*) and embryonic nervous system (*blue*) when directly comparing isolated 4tU-labeled mRNAs with each other. The red bars represent numbers from the reference gene list

We searched for protein classes that are overrepresented among the genes enriched in adult brain versus embryonic CNS by comparing to the reference gene list (Fig. 7a, for *p*-values see Additional file 3). In zebrafish adult differentiating neurons, we found that various neurotransmitter receptors (see [26, 31, 32]) and neuropeptides [33], implicated in processes both during neuronal development and neuronal function, showed enrichment, while transcription factors were less represented than expected when compared to the reference gene list. Further, various types of channels, such as calcium channels and sodium channels, were overrepresented. Calcium signaling is not only important for neuronal proliferation and migration, but also for differentiation by regulating neurotransmitter phenotype, dendritic morphology and axonal growth and guidance (see [34]).

We also examined protein pathways and compared their calculated overrepresentation to the reference gene list. Some pathways were significantly overrepresented (padj < 0.05, Bonferroni correction) during adult neuronal differentiation compared to embryonic neuronal differentiation processes. Interestingly, in adult brain, metabotropic and ionotropic glutamate receptor (GluR) pathways were significantly overrepresented (Fig. 7b, green columns) within adult enriched genes over embryo enriched genes when compared to the reference gene list (red). Further, GABA-B receptor II signaling and muscarinic acetylcholine receptor (AchR) signaling were enriched in adult transgene-expressing cells suggesting that during adult neuronal differentiation, neurotransmitter pathways, most known for their function during neuronal signaling in mature and functional neurons, may play an important role during neuronal differentiation and migration of post-mitotic neurons. Alternatively, it is possible that differentiating neurons in the adult brain start to express mature neuronal genes at earlier steps in the pathway of neuron development than differentiating neurons in the embryonic brain. Importantly, we conclude that the difference between embryonic and adult differentiating neurons is bona fide, and not due to sample contamination from surrounding tissue. This conclusion is based on the significant differences in the comparisons between purified samples and total RNA, which suggest that adult differentiating cells express more transcription factors than are expressed in the whole brain (Figs. 2 and 3).

Pathways represented more frequently (padj < 0.05, Bonferroni correction) than expected in embryonic differentiating neurons (vs adult) include Hedgehog, Notch, Cadherin and Wnt signaling pathways, all of which are critical for neuronal differentiation in the developing embryo [2, 4, 35]. For instance, *lhx2a* and *lhx2b*, which are enriched in embryonic 4tU-expressing cells compared to adult brain, are important for neuronal differentiation in the thalamus by regulating Wnt signaling [36]. We conclude that different pathways are used during embryonic and adult neuronal differentiation. Our results are important because they identify genes to target for more detailed analyses of single gene functions to fully understand the molecular processes involved, especially during neuronal differentiation in the adult brain.

## Conclusions

4tU-labeling identified the transcriptome of differentiating neurons in the adult brain and the embryonic zebrafish nervous system. Some protein classes, such as general binding proteins and in particular transcription factors are overrepresented in embryonic and adult 4tU-labeled mRNA when compared to total mRNA. We verified co-expression of HA-Uprt-mCherry protein with enriched or depleted genes tested by in-situ hybridization and immunohistochemistry. Interestingly, we found that different pathways are overrepresented in adult brain when compared to embryonic neural development, such as the GluR pathway, while morphogen-related pathways appear to be more prominent during embryonic development than in adults. Our finding that the process of neuronal differentiation differs between embryonic and adult stages should now spur additional studies to more closely describe the underlying mechanisms. These insights will help us understand how neurons differentiate in the adult brain and how this process may be enhanced in cases of acute brain trauma.

## Methods

### Generation of the construct UAS:uprt-mCherry-HA

We generated a fusion protein HA-Uprt-mCherry consisting of a vertebrate version of Uprt, a fluorescent protein mCherry for immediate visualization and an HA-epitope for antibody detection [13]. HA-Uprt with vertebrate-specific codon usage, provided by Chris Q. Doe (University of Oregon), was amplified by PCR to contain attB1 and attB2 sites at the 5’ and 3’ ends, respectively. Recombination was performed using Gateway ® BP clonase (Life Technologies) into pDONR221 to generate pME-HA-UPRT [37]. A 3-way recombination reaction using p5E-4xnr-UAS (obtained from Mary Goll), pME-HA-UPRT, and p3E-mCherry-pA (from the Tol2Kit: http://tol2kit.genetics.utah.edu/index.php/Main_Page) into pDESTTol2pA (also from the Tol2Kit) was performed using LR Clonase (Gateway ®), to generate pDEST-4xnr-UAS-HA-UPRT-mCherry.

### Animal husbandry and lines

Purified DNA was injected into fertilized wild-type (AB) zebrafish eggs at the one-cell stage together with RNA encoding Tol2 transposase to generate stable transgenic zebrafish *Tg(UAS:HA-uprt-mcherry)*, following standard protocols [37]. Zebrafish embryos for the 4tU-labeling experiments were obtained from natural spawning of the transgenic lines *Tg(elavl3:Gal4-VP16*_*413–470*_), see *psi1Tg* (zfin.org) and *Tg(UAS:uprt-mcherry-HA)*. Fish were staged by hours post fertilization (hpf) at 28.5 °C [38]. All procedures were carried out under an approved protocol with the University of Oregon Animal Care and Use Committee.

### In situ hybridization and immunohistochemistry

RNA in situ hybridization and immunohistochemistry on whole *Tg(elavl3:Gal4-VP16*_*413–470*_);*Tg(UAS:HA-uprt-mcherry)* embryos and on brain cryostat sections (16 um) were carried out according to standard protocols [39]. The following antibodies were used: anti-HA.11 (clone 16B12, 1:500, abcam), rabbit anti-HA (1:500, Bethyl Laboratories), anti-Elavl3 (ab78467, 1:100, abcam), anti-Elavl (formerly known as anti-HuC/D 16A11, 1:40, University of Oregon), anti-GFAP (zrf-1, 1:100, Zebrafish International Resource Center) and anti-PCNA (1:500, Dako). Primary antibodies were revealed using secondary antibodies coupled to Alexa-Fluor dyes (goat anti-rabbit, goat anti-mouse [H + L], IgG1, IgG2a; 1: 500, Life Technologies) or coupled to horse radish peroxidase (HRP, 1:200, Jackson ImmunoResearch).

### Imaging

Embryos were scored and imaged on an inverted Nikon TU-2000 microscope with an EZ-C1 confocal system (Nikon) or viewed with a Zeiss Axioplan2 microscope and photographed with a Zeiss AxioCam MRc5 camera.

### 4tU delivery in embryos and adult zebrafish and collection of tissue

We incubated *Tg(elavl3:Gal4-VP16*_*413–470*_);*Tg(UAS:HA-uprt-mcherry)* embryos from 22 hpf to 26 hpf in 4tU [29] at a final concentration of 1 mM 4tU/DMSO solution in E3 embryo medium for 4 h in the dark. Whole embryos were euthanized and processed for RNA isolation. For adult experiments, we injected 4tU/DMSO solution at a concentration of 25 mg/mL per 100 mg of fish, mixed with corn oil, into the abdominal cavity of adult 9 month old transgenic female and male zebrafish and let the fish recover in tank water for 4 h. After euthanizing the fish, we recovered the brains and processed them for RNA isolation.

### RNA isolation and affinity purification of thio-labeled mRNA

We isolated total RNA from about 250 embryos or 10 adult brains for each sample, homogenized the tissue in TRIzol (LifeTechnologies) and purified RNA according to the TRIzol protocol. After quantification (Qubit), we diluted the RNA to 0.5 ng/uL and kept 1 uL as total RNA reference. Biotin-Thiol coupling and Streptavidin purification were performed according to published protocols [29]. We analyzed RNA quantity and quality using the fragment analyzer software PROSize.

### First strand synthesis and RNAseq library preparation

Equivalent amounts of streptavidin-purified RNA and total RNA that had been saved directly after the RNA isolation step, were processed using the SMARTer Ultra Low Input mRNA for Illumina Sequencing kit (Clontech). Samples were amplified between 12 and 15 cycles and the cDNA was eluted from magnetic beads in 12 uL water. We used 5 uL from each sample for the “tagmentation” reaction of the Nextera XT DNA sample preparation kit (Illumina). Index primers (Nextera XT Index Kit, Illumina) were added and libraries from four separate RNA samples were mixed at equal concentrations and sequenced in one lane of an Illumina HiSeq 2000 sequencer (Center for Genome Research and Biocomputing Core Facility, OSU) resulting in 100 base pair (bp) single end reads. We sequenced two biological replicates of each purified and total mRNA set, and analyzed the data accordingly. See flow-chart shown in Additional file 1.

### Data analysis

Sequenced reads were filtered for sequences that passed the Illumina chastity filter, had all adapters removed, and were quality trimmed, resulting in sequences with at least 25 bp length, using CutAdapt [40] and Trimmomatic [41], using the parameters SLIDINGWINDOW:5:20 LEADING:20 MINLEN:25. Reads were aligned against the zebrafish genome assembly version Zv9 and annotated using the gene transfer annotation file (GTF) Danio_rerio.Zv9.78 using the splice-site aware program GSNAP [42]. Reads aligning to exons were counted by HTSeq [43], using the “intersection-strict” mode. Only protein-coding genes were considered (BioMart, [44]) and analyzed using DESeq2 [45], using default parameters [46]. Gene Ontology was analyzed using the Panther Classification System [27], using the settings *p*-value < 0.05 and true Bonferroni correction.

### Availability of supporting data

The data set supporting the results of this article are available in the LabArchives repository DOI 10.6070/H4QC01HW (https://mynotebook.labarchives.com/doi/MTMzMjM3LjB8MTAyNDkwLzEwMjQ5MC9Ob3RlYm9vay8zOTU4MDY1MzAzfDMzODIxNy4w/10.6070/H4QC01HW) and within the article and its additional files 1, 2, 3, 4, 5, 6, available as Adobe pdf file or excel spreadsheets (xlsx file format).

## Additional files

Additional file 1:
**Flow-chart of the method 4tU-labeling.** (PDF 303 kb) Additional file 2:
**DESeq2 results showing a comparison between labeled mRNA and total mRNA from adult brain with padj < 0.1.** (XLSX 127 kb) Additional file 3:
**Original Panther analysis tool data tables showing values and** ***p*** **-values for overrepresentation data sets.** (XLSX 21 kb) Additional file 4:
**List of genes expressed in the nervous system obtained from ZebrafishMine (http://www.zebrafishmine.org/begin.do) and Panther Classification system [** 27 **].** (XLSX 146 kb) Additional file 5:
**DESeq2 results showing a comparison between labeled mRNA and total mRNA from embryos with padj < 0.1.** (XLSX 83 kb) Additional file 6:
**DESeq2 results showing a comparison between labeled mRNA from adult brain and labeled mRNA from embryos with padj < 0.05.** (XLSX 468 kb)

## Abbreviations

4tU: 4-thiouracil
CNS: Central nervous system
dHab: Dorsal Habenula
DiV: Diencephalic ventricle
dTel: Dorsal Telencephalon
EG: Eminentia granularis
HA: Hemaglutinin tag
Hab: Habenula
IHC: Immunohistochemistry
ISH: In situ hybridization
LCa: Lobus caudalis cereblli
PCNA: Proliferating cell nuclear antigen
PGZ: Periventricular grey zone
TeO: Optic tectum
TelV: Telencephalic ventricle
TF: Transcription factor
uprt: uracil-phosphoribosyl-transferase
vHab: ventral Habenula
vTel: ventral Telencephalon
VOT: Ventrolateral optic tract
