# Supplementary figures and images for: Transcriptomes of post-mitotic neurons identify the usage of alternative pathways during adult and embryonic neuronal differentiation
Source: BMC Genomics. 2015 Dec 23;16:1100. doi: 10.1186/s12864-015-2215-8 (PMC4690400; doi:10.1186/s12864-015-2215-8)

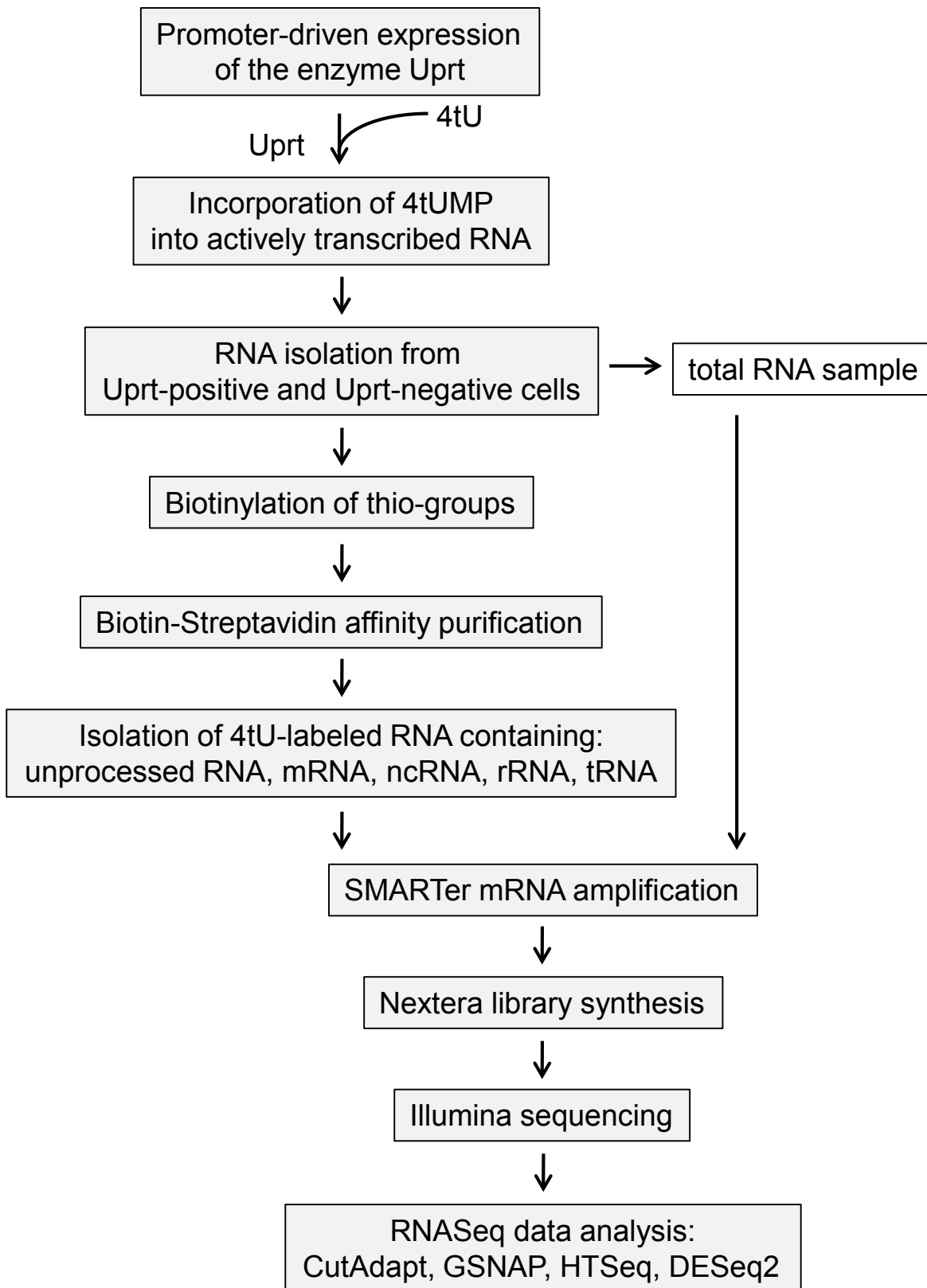

Supplement: Additional file 1: — Flow-chart of the method 4tU-labeling. (PDF 303 kb) [file 12864_2015_2215_MOESM1_ESM.pdf]
